# Supplementary material for: Synergistic patient factors are driving recent increased pediatric urgent care demand
Source: PLOS Digit Health. 2024 Aug 22;3(8):e0000572. doi: 10.1371/journal.pdig.0000572 (PMC11340883; doi:10.1371/journal.pdig.0000572)
Supplement: S1 Fig — Histogram of untransformed length of stay in minutes (A) compared to the more normalized (although still right skewed) distribution after log-10 transformation (B). (DOCX) [file pdig.0000572.s001.docx]

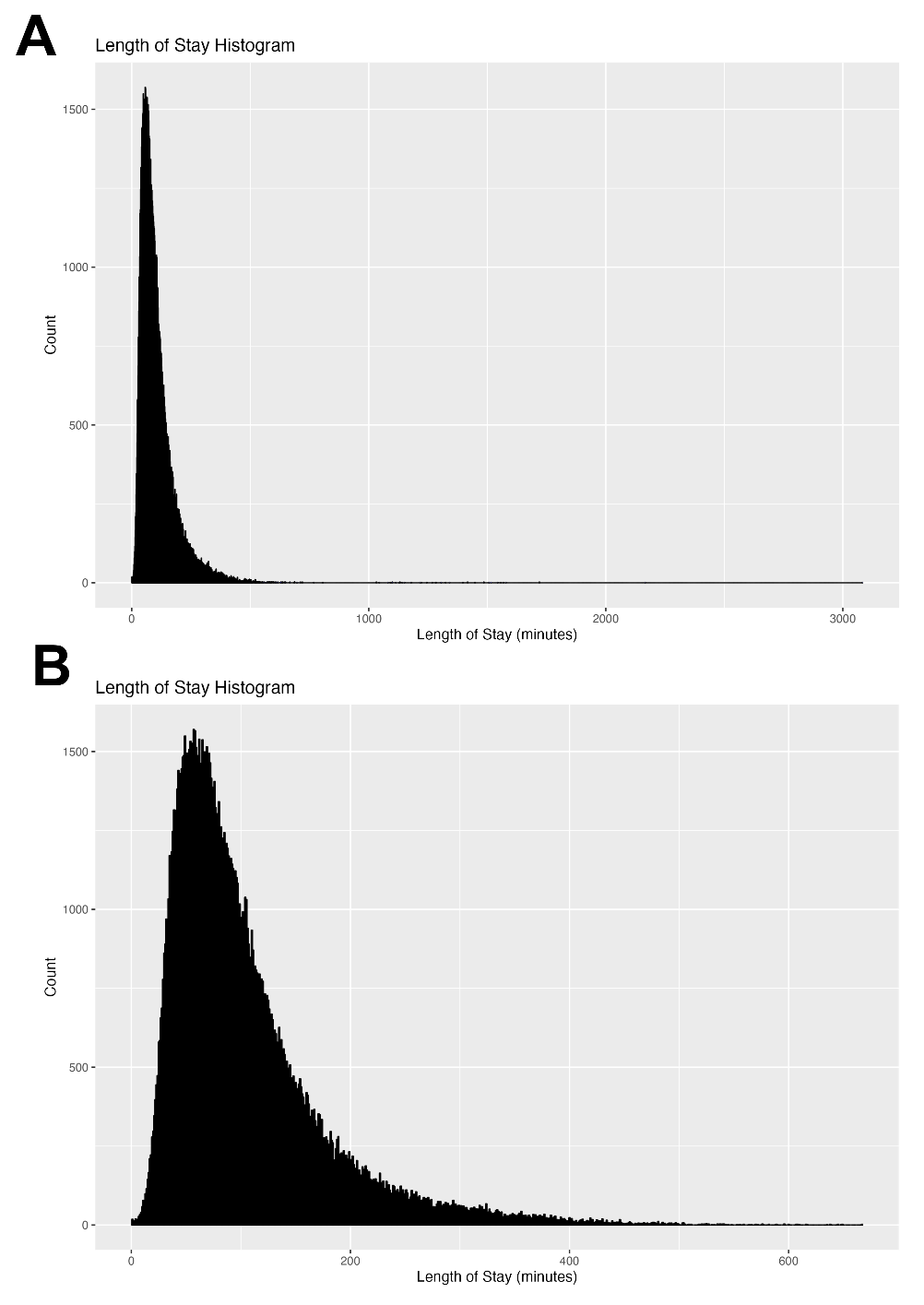


**S1 Fig.** Histogram of untransformed length of stay in minutes (A) compared to the more normalized (although still right skewed) distribution after log-10 transformation (B).
