# Supplementary material for: Synergistic patient factors are driving recent increased pediatric urgent care demand
Source: PLOS Digit Health. 2024 Aug 22;3(8):e0000572. doi: 10.1371/journal.pdig.0000572 (PMC11340883; doi:10.1371/journal.pdig.0000572)
Supplement: S2 Fig — A linear regression model was trained on 80% of the data, then predicted (on the 20% testing dataset, having not been exposed to this set before) length of stay (A) with an r-squared value of 0.838 with an root mean squared error (RMSE) of 0.108. The actual length of stay was overestimated for longer visits by the linear regression model (B). A random forest model was also trained on 80% of the data, then predicted (on the 20% testing dataset, having not been exposed to this set before) length of stay (C) with an r-squared value of 0.830 and an RMSE of 0.133. The actual length of stay was underestimated for longer visits by the random forest model (D). The relative importance of features the random forest model used were (in descending order): time of day discharged, time of day registered, a presentation in the year 2022, age (younger = longer), a discharge disposition home, a “head and neck” category chief complaints, and a presentation in the year 2021 (E). (DOCX) [file pdig.0000572.s002.docx]

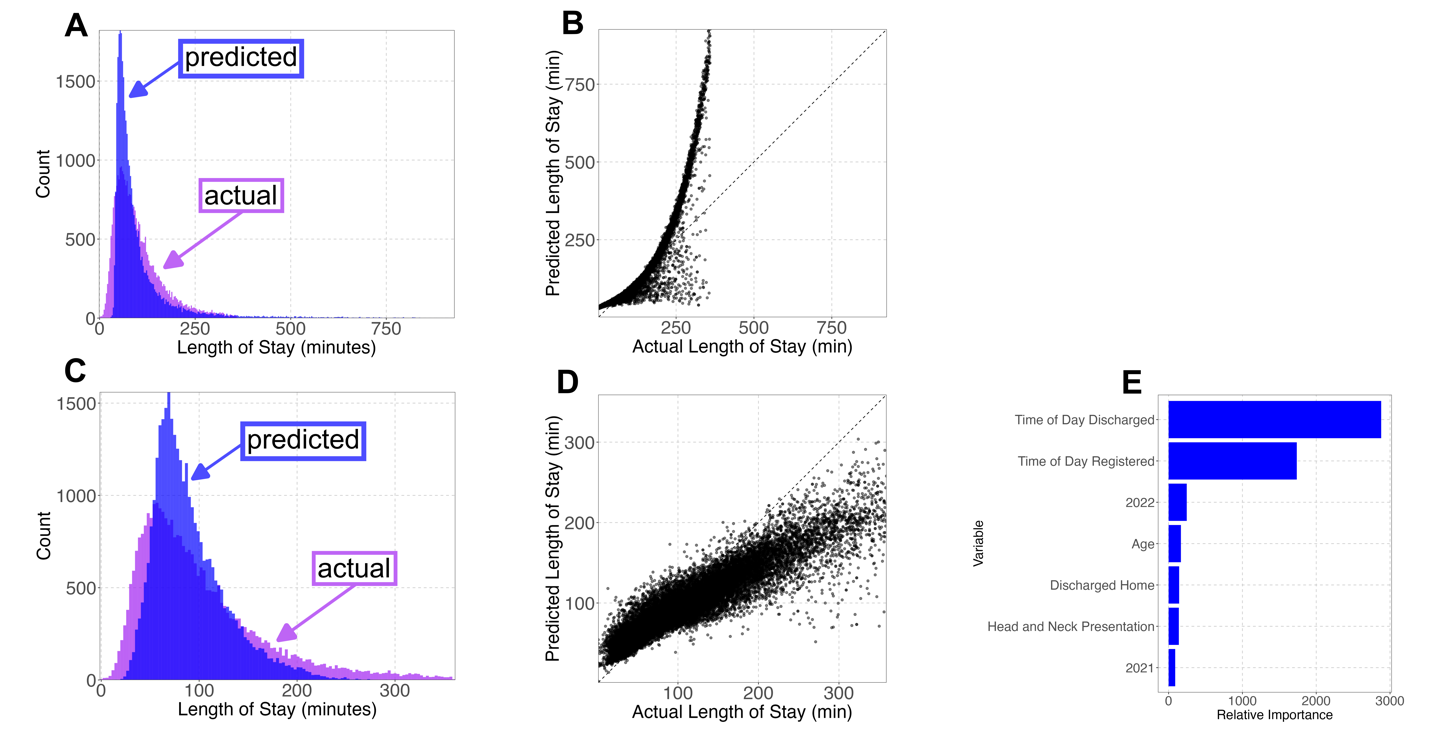


**S2 Fig**. **Comparison of machine learning models to predict length of stay.** A linear regression model was trained on 80% of the data, then predicted (on the 20% testing dataset, having not been exposed to this set before) length of stay (A) with an r-squared value of 0.838 with an root mean squared error (RMSE) of 0.108. The actual length of stay was overestimated for longer visits by the linear regression model (B). A random forest model was also trained on 80% of the data, then predicted (on the 20% testing dataset, having not been exposed to this set before) length of stay (C) with an r-squared value of 0.830 and an RMSE of 0.133. The actual length of stay was underestimated for longer visits by the random forest model (D). The relative importance of features the random forest model used were (in descending order): time of day discharged, time of day registered, a presentation in the year 2022, age (younger = longer), a discharge disposition home, a “head and neck” category chief complaints, and a presentation in the year 2021 (E).
