# Supplementary material for: Synergistic patient factors are driving recent increased pediatric urgent care demand
Source: PLOS Digit Health. 2024 Aug 22;3(8):e0000572. doi: 10.1371/journal.pdig.0000572 (PMC11340883; doi:10.1371/journal.pdig.0000572)
Supplement: S1 File — (DOCX) [file pdig.0000572.s003.docx]

**RStudio Session Info**

R version 4.3.1 (2023-06-16)

Platform: aarch64-apple-darwin20 (64-bit)

Running under: macOS Sonoma 14.0

locale:

[1] en_US.UTF-8/en_US.UTF-8/en_US.UTF-8/C/en_US.UTF-8/en_US.UTF-8

Time zone: America/Toronto

tzcode source: internal

Attached base packages:

stats

graphics

grDevices

utils

datasets

methods

base

All Attached Packages by Version:

| **Package** | **Version** |
| --- | --- |
| knitr | 1.45 |
| ranger | 0.15.1 |
| vip | 0.4.1 |
| yardstick | 1.2.0 |
| workflowsets | 1.0.1 |
| workflows | 1.1.3 |
| tune | 1.1.2 |
| rsample | 1.2.0 |
| recipes | 1.0.8 |
| parsnip | 1.1.1 |
| modeldata | 1.2.0 |
| infer | 1.0.5 |
| dials | 1.2.0 |
| scales | 1.2.1 |
| broom | 1.0.5 |
| tidymodels | 1.1.1 |
| e1071 | 1.7.13 |
| reshape2 | 1.4.4 |
| ggrepel | 0.9.4 |
| janitor | 2.2.0 |
| skimr | 2.1.5 |
| readxl | 1.4.3 |
| officer | 0.6.3 |
| glue | 1.6.2 |
| DT | 0.3 |
| magrittr | 2.0.3 |
| lubridate | 1.9.3 |
| forcats | 1.0.0 |
| stringr | 1.5.0 |
| dplyr | 1.1.3 |
| purrr | 1.0.2 |
| readr | 2.1.4 |
| tidyr | 1.3.0 |
| tibble | 3.2.1 |
| ggplot2 | 3.4.4 |
| tidyverse | 2.0.0 |
| stats | 4.3.1 |
| graphics | 4.3.1 |
| grDevices | 4.3.1 |
| utils | 4.3.1 |
| datasets | 4.3.1 |
| methods | 4.3.1 |
| base | 4.3.1 |
