# Supplementary material for: Synergistic patient factors are driving recent increased pediatric urgent care demand
Source: PLOS Digit Health. 2024 Aug 22;3(8):e0000572. doi: 10.1371/journal.pdig.0000572 (PMC11340883; doi:10.1371/journal.pdig.0000572)
Supplement: Attachment — Submitted filename: PLOS Digital Health Replies to Reviewers_2024-06-20.docx [file pdig.0000572.s004.docx]

**Replies to Reviewers for Manuscript PDIG-D-24-00160
“Synergistic patient factors are driving recent increased pediatric urgent care demand”
PLOS Digital Health**

[Reviewer comments in **bold**, author replies listed in plain text below]

**Additional Editor Comments (if provided):**

(Please note that some replies to the Editor are highlighted points also asked by specific reviewers. For clarity, these points to the Editor and the analogous points from the reviewers are harmonized/identical.)

**Introduction:
It is recommended to include in the introduction more information regarding specific background (machine learning models)**

- We have added a paragraph in the Introduction section discussing machine learning models in this context that reads:
- “Patterns in routinely collected clinical data are difficult to uncover. Increasing availability of large multiparameter clinical databases has driven the adoption of machine learning (ML) techniques for analysis of multiparameter clinical data {Reel et al}. One powerful technique to identify patterns that are not obvious when interpreted by a human is machine learning (ML). ML is the application of algorithms to data analysis in such a way that the accuracy of the analysis can be improved by automated iteration {chen et al}. The computational power of modern computers facilitates the use of ML algorithms to discern patterns in clinical data that are complex and non-linear {Chen et al}.”
- Reel, P. S., Reel, S., Pearson, E., Trucco, E. & Jefferson, E. Using machine learning approaches for multi-omics data analysis: A review. *Biotechnol Adv* **49**, 107739 (2021). <https://doi.org:10.1016/j.biotechadv.2021.107739>
- Chen, J. H. & Asch, S. M. Machine Learning and Prediction in Medicine - Beyond the Peak of Inflated Expectations. *N Engl J Med* **376**, 2507-2509 (2017).

**Methodology.
Study Design: Specify whether the study is observational, retrospective, prospective, experimental, etc.**

- We have added to the study design section that this is a retrospective cohort study.

**Population and Sample: Describe the target population and the sample selection process. Include inclusion and exclusion criteria.**

- We have added a detailed description of the target population and identification process including inclusion and exclusion criteria to the Methods section that reads:
- “The study cohort consisted of all consecutive patient encounters obtained from our local healthcare centre’s pediatric urgent care NACRS retrospective monitoring data between April 1, 2006 to Dec 31, 2022. The inclusion criteria were patients under 18 years of age, seen at the urgent care centre within the study timeline, regardless of healthcare coverage status. Exclusion criteria were any of: any incomplete demographics or clinical details in the extracted fields listed above or the visit was begun more than 60 min before the scheduled opening time (28 visits), the patient was discharged prior to the scheduled opening time (13 visits), the patient left more than 15 hours after the scheduled opening time (which is midnight, 124 visits), and if the length of stay was greater than 1000 min (that is 16 hours and 40 minutes; 13,651 visits). Given that our urgent care centre is only staffed for up to about 12 hours (and then is closed overnight), records with very long visit times represented patients who were missed as being recorded discharged in the health record and then coded as discharged at the start of the next day, which is a known clerical work around used in our system.”

**Data Collection: Briefly outline the data collection methods (e.g., medical record review, surveys, interviews).**

- We have briefly outlined the data collection methods as follows:
- “The data was collected from the electronic health record database (EHR) for our hospital centre that is used for mandatory reporting to the Ministry of Health. This data is reviewed by our Decision Support teams prior to reporting, so it is of relatively high quality. Thus, we extracted only data fields from the EHR that are cleaned and reported to the Ministry.”

**Variables: Identify the primary variables to be analyzed (e.g., demographic characteristics, waiting times, reasons for consultation).**

- We have added to the Methods section these details as follows:
- “Variables analyzed included: basic patient demographics (age, sex, primary care status yes/no/unknown), chief complaint, triage urgency (using the standardized Canadian Triage and Acuity Scale, CTAS), date and time of registration, time of discharge, discharge diagnosis, and discharge destination (home/admission/left without being seen).”

**Data Analysis: Indicate the statistical analysis methods to be employed for interpreting the data (e.g., bivariate analysis, multivariate regression). It is not clear and need to be justified, why the authors did not include visits longer than 1000 min, could longer visits affect the results?**

- We have updated the statistical analysis section of the paper to include:
- “We used univariate linear regression as a baseline model.”
- We have also explained in detail the rationale for exclusion of visits longer than 1000 minutes:
- “Given that our urgent care centre is only staffed for up to about 12 hours (and then is closed overnight), records with very long visit times represented patients who were missed as being recorded discharged in the health record and then coded as discharged at the start of the next day, which is a known clerical work around used in our system.”

**Why did the linear regression model have tendency to overestimate long visits. Was this the reason why visits above 1000 minutes were excluded from the analysis?**

- We have added the following paragraph to the discussion section:
- “It is likely that the linear regression model tended to overestimate length for long visits as the visit length data was strongly left skewed, even when log-10 transformed (Figure S1), thus the longer visits acted as outliners and were less accurately modelled. Linear regression models are known to be less accurate for outliers and skewed datasets. Despite this limitation, the large majority of visits were accurately predicted by the linear regression model (Figure S2A), but the longer less common visit lengths were overestimated (Figure S2B) by this model as the longer visits deviate from the normal. Thus, the longer the visit, the less accurate this relatively simple model can predict.”
- Please see the previous reply for the reason why visits above 1000 minutes were excluded.

**Please consider as confounding factors staffing fluctuations and quantity of paperwork, those factors play a role on the length of stay. If data are not available, those factors could be considered a limitation of the study**

- We have added to the limitations paragraph of the discussion:
- “Another limitation is that workload fluctuations, including nursing ratios and paperwork quantity, may have played a role in determining patient length of stay, although this data is not tracked at our centre. It would be helpful if these factors were added to the current reporting requirements, so these could be accurately evaluated in future studies.”

**Results
It is important to include in the explanation of limitations the validity of studying only one center in terms of demography, center capacity in electronic health records**

- We have added to the limitations section the following:
- “This study was limited to a single centre in a medium size city serving a large regional population in Canada, which limits generalizability for some findings. However, the trends seen in patient characteristics and factors contributing to increased care needs would be readily applicable to many similar sized centres in North America. Another limitation is that this study relies on electronic health records, which are imperfect and can bias data in unexpected ways from incomplete or inaccurate recording.”

**Discussion
Expand the discussion with machine learning models in action. Please be more explicit about how machine learning facilitated this study because it seems that similar analysis can be done without the help of a machine learning software**

- We have added the following section to the discussion:
- “In the present study, ML models were leveraged to analyze an extensive imperfect clinical dataset with numerous variable types. A traditional statistical approach would require simpler modelling that is less effective and less generalizable (as seen in the differences between models in Figure S2). The automation, efficiency, and scalability of ML models are strengths, especially for large data sets such as this.”

**Please include in the discussion about the importance of machine learning techniques to obtain these results. Discuss how random forest model can be used in the future in medicine for prediction models.**

- We appreciate the opportunity to improve the machine learning aspects of this study. In addition to the addition to the discussion in the point directly above, which partially addresses this point, we have added to the discussion section:
- “Random forest models specifically are ideal for large organized digital health datasets such as the one analyzed in this study. Random forest models are: relatively efficient compared to more complex models such as neural networks, less prone to overfitting, and amenable to missing data (common concerns in human health data research). Thus, random forest models provide an excellent “out-of-the-box” ML model to use for clinical data analysis.”

**Reviewer #1:**

**The findings are based on data from one center, limiting generalizability. It does not account for changes in local population demographics, center’s capacity such as staffing or resources and introduction and evolution of Electronic Health Records (EHR).**

- We have added to the limitations section the following:
- “This study was limited to a single centre in a medium size city serving a large regional population in Canada, which limits generalizability for some findings. However, the trends seen in patient characteristics and factors contributing to increased care needs would be readily applicable to many similar sized centres in North America. Another limitation is that this study relies on electronic health records, which are imperfect and can bias data in unexpected ways from incomplete or inaccurate recording.”

**The study lacks a clear discussion on the clinical impacts of the observed trends, leaving the practical significance of the findings ambiguous.**

- We have added as the second paragraph of the discussion:
- “The findings help to identify current deficiencies in the health care system that could be targeted for change. Declining access to primary care means patients may wait longer to seek medical care and may thus be presenting in a sicker state. Identifying an increase in infectious presentations means that preventive measures such as public health campaigns could target these needs. Additionally, the increase in patients with more complex needs may mean that longer visits are a new reality for urgent care centres.”

**The practical benefits of predicting visits to be in 2022 are unclear.**

- We have added to the discussion section:
- “Using a machine learning model to predict more recent visits is one approach to identify patient factors that have changed from previous years. A similar approach has been previously used to predict emergency department visits {Gafni-Pappas et al}, and hospital admissions and outpatient visits {Habbous et al}. Since 2022 was the most recent year for which we had available high-quality data, and because the trends explored in Figures 1-3 were strikingly different in 2022, we used 2022 visits as our outcome for this part of the analysis. We aimed to discover what patient factors were different in 2022 that could explain this difference. Thus, we built a model to identify important but subtle statistical differences between 2022 and previous years that would be unlikely to be evident from simple trend analysis.”
- Gafni-Pappas G, Khan M. Predicting daily emergency department visits using machine learning could increase accuracy. Am J Emerg Med. 2023;65:5-11.
- Habbous S, Lambrinos A, Petersen S, Hellsten E. The effect of the COVID-19 pandemic on hospital admissions and outpatient visits in Ontario, Canada. Ann Thorac Med. 2023;18(2):70-78.

**It is unclear why the authors filter out the visits longer than 1000 minutes.**

- We have now explained in detail the rationale for exclusion of visits longer than 1000 minutes:
- “Given that our urgent care centre is only staffed for up to about 12 hours (and then is closed overnight), records with very long visit times represented patients who were missed as being recorded discharged in the health record and then coded as discharged at the start of the next day, which is a known clerical work around used in our system.”

**Reviewer #2:**

**Firstly, I recommend presenting the Methods part with the Results and Discussion**

- We have moved the Methods section to before the Results. We verified that PLOS Digital Health recommends the Methods section to follow the Discussion but it is acceptable for this journal to have the Methods section before the Results.

**Also, I recommend amending the Introduction with more information regarding specific background (machine learning models).**

- We have added a paragraph in the Introduction section discussing machine learning models in this context that reads:
- “Patterns in routinely collected clinical data are difficult to uncover. Increasing availability of large multiparameter clinical databases has driven the adoption of machine learning (ML) techniques for analysis of multiparameter clinical data.{Reel et al} One powerful technique to identify patterns that are not obvious when interpreted by a human is machine learning (ML). ML is the application of algorithms to data analysis in such a way that the accuracy of the analysis can be improved by automated iteration.{chen et al} The computational power of modern computers facilitates the use of ML algorithms to discern patterns in clinical data that are complex and non-linear.{Chen et al}”
- Reel, P. S., Reel, S., Pearson, E., Trucco, E. & Jefferson, E. Using machine learning approaches for multi-omics data analysis: A review. *Biotechnol Adv* **49**, 107739 (2021). <https://doi.org:10.1016/j.biotechadv.2021.107739>
- Chen, J. H. & Asch, S. M. Machine Learning and Prediction in Medicine - Beyond the Peak of Inflated Expectations. *N Engl J Med* **376**, 2507-2509 (2017).

**Next, I believe that the Discussion part requires expanding to include more examples of such models in action**

- We appreciate the opportunity to improve the machine learning aspects of this study. We have added to the discussion section:
- “In the present study, ML models were leveraged to analyze an extensive imperfect dataset with numerous variable types. A traditional statistical approach would require simpler modelling that is less effective and less generalizable (as seen in the differences between models in Figure S2). The automation, efficiency, and scalability of ML models are strengths, especially for large data sets such as this.”
- “Random forest models specifically are ideal for large organized digital health datasets such as the one analyzed in this study. Random forest models are: relatively efficient compared to more complex models such as neural networks, less prone to overfitting, and amenable to missing data (common concerns in human health data research). Thus, random forest models provide an excellent “out-of-the-box” ML model to use for clinical data analysis.”
- “The findings help to identify current deficiencies in the health care system that could be targeted for change. Declining access to primary care means patients may wait longer to seek medical care and may thus be presenting in a sicker state. Identifying an increase in infectious presentations means that preventive measures such as public health campaigns could target these needs. Additionally, the increase in patients with more complex needs may mean that longer visits are a new reality for urgent care centres.”
- “Using a machine learning model to predict more recent visits is one approach to identify patient factors that have changed from previous years. A similar approach has been previously used to predict emergency department visits {Gafni-Pappas et al}, and hospital admissions and outpatient visits {Habbous et al}. Since 2022 was the most recent year for which we had available high-quality data, and because the trends explored in Figures 1-3 were strikingly different in 2022, we used 2022 visits as our outcome for this part of the analysis. We aimed to discover what patient factors were different in 2022 that could explain this difference. Thus, we built a model to identify important but subtle statistical differences between 2022 and previous years that would be unlikely to be evident from simple trend analysis.”
- Gafni-Pappas G, Khan M. Predicting daily emergency department visits using machine learning could increase accuracy. Am J Emerg Med. 2023;65:5-11.
- Habbous S, Lambrinos A, Petersen S, Hellsten E. The effect of the COVID-19 pandemic on hospital admissions and outpatient visits in Ontario, Canada. Ann Thorac Med. 2023;18(2):70-78.

**Reviewer #3:**

**Major comment 1: The study does not account for very important factors influencing the length of stay in acute care services, such as evolving documentation practices over time (more paperwork) and staffing fluctuations (bigger patient to provider ratios), particularly notable in the aftermath of the COVID-19 pandemic. These variables should be incorporated into the analysis as potential confounding factors. If data on these aspects are unavailable, authors should acknowledge these as limitations of the study.**

- We have added to the limitations paragraph of the discussion:
- “Another limitation is that workload fluctuations, including nursing ratios and paperwork quantity, may have played a role in determining patient length of stay, although this data is not tracked at our centre. It would be helpful if these factors were added to the current reporting requirements, so these could be accurately evaluated in future studies.”

**Major comment 2: Patient should be more explicit about how machine learning facilitated this study because it seems that similar analysis can be done without the help of a machine learning software.**

- We appreciate the opportunity to improve the machine learning aspects of this study.
- We have added a paragraph in the Introduction section discussing machine learning models in this context that reads:
- “Patterns in everyday clinical data such as the differential are difficult to uncover, since clinicians mostly view information on a patient-by-patient basis. Increasing availability of large multiparameter datasets has driven the adoption of machine learning (ML) techniques for analysis of multiparameter clinical data.{Reel et al} ML can identify patterns that are not obvious when interpreted by a human. Machine learning (ML) is the application of algorithms to data analysis in such a way that the accuracy of the analysis can be improved by automated iteration.{chen et al} The computational power of modern computers facilitates the use of ML algorithms to use automated trial-and-error style evaluation to discern patterns in clinical data that are complex and non-linear.{Chen et al}”
- Reel, P. S., Reel, S., Pearson, E., Trucco, E. & Jefferson, E. Using machine learning approaches for multi-omics data analysis: A review. *Biotechnol Adv* **49**, 107739 (2021). <https://doi.org:10.1016/j.biotechadv.2021.107739>
- Chen, J. H. & Asch, S. M. Machine Learning and Prediction in Medicine - Beyond the Peak of Inflated Expectations. *N Engl J Med* **376**, 2507-2509 (2017).
- We have added to the discussion section:
- “In the present study, ML models were leveraged to analyze an extensive imperfect dataset with numerous variable types. A traditional statistical approach would require simpler modelling that is less effective and less generalizable (as seen in the differences between models in Figure S2). The automation, efficiency, and scalability of ML models are strengths, especially for large data sets such as this.”
- “The findings help to identify current deficiencies in the health care system that could be targeted for change. Declining access to primary care means patients may wait longer to seek medical care and may thus be presenting in a sicker state. Identifying an increase in infectious presentations means that preventive measures such as public health campaigns could target these needs. Additionally, the increase in patients with more complex needs may mean that longer visits are a new reality for urgent care centres.”
- “Using a machine learning model to predict more recent visits is one approach to identify patient factors that have changed from previous years. A similar approach has been previously used to predict emergency department visits {Gafni-Pappas et al}, and hospital admissions and outpatient visits {Habbous et al}. Since 2022 was the most recent year for which we had available high-quality data, and because the trends explored in Figures 1-3 were strikingly different in 2022, we used 2022 visits as our outcome for this part of the analysis. We aimed to discover what patient factors were different in 2022 that could explain this difference. Thus, we built a model to identify important but subtle statistical differences between 2022 and previous years that would be unlikely to be evident from simple trend analysis.”
- Gafni-Pappas G, Khan M. Predicting daily emergency department visits using machine learning could increase accuracy. Am J Emerg Med. 2023;65:5-11.
- Habbous S, Lambrinos A, Petersen S, Hellsten E. The effect of the COVID-19 pandemic on hospital admissions and outpatient visits in Ontario, Canada. Ann Thorac Med. 2023;18(2):70-78.

**Minor comment 1: Why did the linear regression model have tendency to overestimate long visits. Was this the reason why visits above 1000 minutes were excluded from the analysis?   Was ever considered including patients who had visits of more than 1000 minutes? I am wondering how including those patients would affect the results?**

- We have explained in detail the rationale for exclusion of visits longer than 1000 minutes:
- “Given that our urgent care centre is only staffed for up to about 12 hours (and then is closed overnight), records with very long visit times represented patients who were missed as being recorded discharged in the health record and then coded as discharged at the start of the next day, which is a known clerical work around used in our system.”

**Minor comment 2: Please include in the discussion about the importance of machine learning techniques to obtain these results. Discuss how random forest model can be used in the future in medicine for prediction models.**

- We appreciate the opportunity to improve the machine learning aspects of this study. In addition to the addition to the additions to the discussion in Major Comment 2 above, which partially address this point, we have added to the discussion section:
- “Random forest models specifically are ideal for large organized digital health datasets such as the one analyzed in this study. Random forest models are: relatively efficient compared to more complex models such as neural networks, less prone to overfitting, and amenable to missing data (common concerns in human health data research). Thus, random forest models provide an excellent “out-of-the-box” ML model to use for clinical data analysis.”

**Reviewer #4:**

**The methods presented in the abstract are not sufficiently clear. While a full dissertation on the methodology is not required, it is recommended to provide a brief methodological description. The methodology of the study is not clearly identified in the methods section.**

- We have rewritten the methods portion of the abstract and it now reads:
- “The dataset for this retrospective cohort study was obtained from our local healthcare centre’s national reporting data for pediatric urgent care visits from 2006 to 2022. Variables analyzed included: basic patient demographics, chief complaint, triage urgency, date and time of registration/discharge, discharge diagnosis, and discharge destination. Statistical analysis of non-linear trends was summarized by locally estimated scatterplot smoothing splines. For machine learning, we used the tidymodels R package. Models were validated in training using k-fold cross validation with k = 5. We used univariate linear regression as a baseline model. After the data was standardized, correlation and homoscedasticity were evaluated between all parameter permutations.”

**To enhance the introduction of an article, particularly in terms of methodology, it is crucial to include a succinct description of the approaches and techniques to be used for investigating the hypotheses and achieving the study's objectives.**

- We have added the following to the end of the introduction:
- “In this study, we retrospectively analyzed a large longitudinal dataset of pediatric urgent care visits to determine the the factors that have led to the increased pediatric urgent care demand. Time series analysis and machine learning was used to uncover patient factors that changed as demand increased. We hypothesized that patient factors have shifted over time driving the recent increased pediatric urgent care service demand.”
- We have also added a paragraph earlier in the Introduction section discussing machine learning models in this context that reads:
- “Patterns in everyday clinical data such as the differential are difficult to uncover, since clinicians mostly view information on a patient-by-patient basis. Increasing availability of large multiparameter datasets has driven the adoption of machine learning (ML) techniques for analysis of multiparameter clinical data.{Reel et al} ML can identify patterns that are not obvious when interpreted by a human. Machine learning (ML) is the application of algorithms to data analysis in such a way that the accuracy of the analysis can be improved by automated iteration.{chen et al} The computational power of modern computers facilitates the use of ML algorithms to use automated trial-and-error style evaluation to discern patterns in clinical data that are complex and non-linear.{Chen et al}”
- Reel, P. S., Reel, S., Pearson, E., Trucco, E. & Jefferson, E. Using machine learning approaches for multi-omics data analysis: A review. *Biotechnol Adv* **49**, 107739 (2021). <https://doi.org:10.1016/j.biotechadv.2021.107739>
- Chen, J. H. & Asch, S. M. Machine Learning and Prediction in Medicine - Beyond the Peak of Inflated Expectations. *N Engl J Med* **376**, 2507-2509 (2017).

**The following methodological elements could be incorporated into the introduction to make it more comprehensive:**

**Study Design: Specify whether the study is observational, retrospective, prospective, experimental, etc.**

- We have added to the study design section that this is a retrospective cohort study.

**Population and Sample: Describe the target population and the sample selection process. Include inclusion and exclusion criteria.**

- We have added a detailed description of the target population and identification process including inclusion and exclusion criteria to the Methods section that reads:
- “The study cohort consisted of all consecutive patient encounters obtained from our local healthcare centre’s pediatric urgent care NACRS reporting data between April 1, 2006 to Dec 31, 2022. The inclusion criteria were patients under 18 years of age, seen at the urgent care centre within the study timeline, regardless of healthcare coverage status. Exclusion criteria were any of: any incomplete demographics or clinical details in the extracted fields listed above or the visit was begun more than 60 min before the scheduled opening time (28 visits), the patient was discharged prior to the scheduled opening time (13 visits), the patient left more than 15 hours after the scheduled opening time (which is midnight, 124 visits), and if the length of stay was greater than 1000 min (13,651 visits). Given that our urgent care centre is only staffed for up to about 12 hours (and then is closed), records with very long visit times represented patients who were long prior discharged but missed as being recorded as such in the health record and then coded as discharged the next day, which is a known clerical work around used in our system.”

**Data Collection: Briefly outline the data collection methods (e.g., medical record review, surveys, interviews).**

- We have briefly outlined the data collection methods as follows:
- “Data was collected from the electronic health record database (EHR) for our hospital centre that is used for mandatory reporting to the Ministry of Health. This data is reviewed by our Decision Support teams prior to reporting, so we felt would likely be high quality. Thus, we extracted only data fields from the EHR that are also reported to the Ministry.”

**Variables: Identify the primary variables to be analyzed (e.g., demographic characteristics, waiting times, reasons for consultation).**

- We have added to the Methods section these details as follows:
- “Variables analyzed included: basic patient demographics (age, sex, primary care status yes/no/unknown), chief complaint, triage urgency (using the standardized Canadian Triage and Acuity Scale, CTAS), date and time of registration, time of discharge, discharge diagnosis, and discharge destination (home/admission/left without being seen).”

**Data Analysis: Indicate the statistical analysis methods to be employed for interpreting the data (e.g., bivariate analysis, multivariate regression).
For example, it is suggested: "To investigate this, we will employ an observational and retrospective study design, analyzing data collected through NACRS. We will analyze the demographic, clinical, and healthcare system characteristics of patients attending the pediatric urgent care center. Statistical methods will include bivariate and multivariate analyses to identify factors contributing to the increased demand. Our objective is to use high-fidelity NACRS data to model and identify factors contributing to the increased demand for pediatric urgent care at our local urgent care center."**

- We appreciate the specific wording suggested and have updated the statistical analysis section of the paper to include this section:
- “Our objective was to use high-fidelity NACRS data to model and identify factors contributing to the increased demand for pediatric urgent care at our local urgent care center. To investigate this, we employed an observational retrospective study design, analyzing data collected through NACRS. We analyzed the demographic, clinical, and healthcare system characteristics of patients attending the pediatric urgent care centre. Statistical methods included bivariate and multivariate analyses to identify factors contributing to the increased demand.”

**Establishing these considerations is necessary to understand the methodological framework of the article. Although there is good data modeling, it is recommended to include a table that analyzes the factors in the bivariate model with the main variables.**

- We have added this table to the results section individually assessing the factors compared to the main outcome variables addressed in our study:

**Table 2.** Individual comparison of main outcome variables with patient factors.

| **Outcome Variable** | **Factor** | **Statistical Measure** | **Value^a^** | **p-value^b^** |
| --- | --- | --- | --- | --- |
| Visit in 2022 | Length of Stay | t-test | 68.4 | **<1*10^-5^** |
|  | Age | t-test | -1.82 | 6.9*10^-2^ |
|  | Sex | Chi-square | 2.04 | 1.5*10^-1^ |
|  | Acuity (CTAS) | Chi-square | 1097 | **<1*10^-5^** |
|  | Primary care access | Chi-square | 294 | **<1*10^-5^** |
| Length of Stay | Age | Pearson Correlation | 0.638 | **<1*10^-5^** |
|  | Sex | t-test | 3.30 | **9.8*10^-4^** |
|  | Acuity (CTAS) | ANOVA | 10822 | **<1*10^-5^** |
|  | Primary care access | t-test | 4.71 | **<1*10^-5^** |

a, for t-test value is t-statistic, for Chi-square test value is Chi-squared statistic, for Correlation value is rho-squared; b, values in bold if p-value <0.05

*ANOVA, analysis of variance; CTAS, Canadian Triage and Acuity Scale*

Of note, the data was too sparse for the chief complaints to be analyzed by Chi-square or Fisher’s exact test

**It is advised to present the results concisely using standard analysis tables.**

- We have added the following standard tables to the results section comparing data for the relevant variables for visits prior to 2019, visits in 2020/2021 and those in 2022:

**Table 1.** Summary of patient factors across the timeline of the study.

|  | **Visits 2007-2019** | **Visits 2020-2021** | **Visits 2022** |
| --- | --- | --- | --- |
| **Age in years, mean (SD)** | 5.75 (5.16) | 6.03 (5.38) | 5.70 (5.05) |
| **Sex, proportion female** | 0.48 | 0.49 | 0.48 |
| **Acuity (CTAS), mean (SD)** | 3.88 (0.61) | 3.80 (0.63) | 3.73 (0.62) |
| **Lack of primary care access, proportion** | 0.2 | 0.18 | 0.13 |
| **Length of stay in minutes, mean (SD)** | 95.4 (63.0) | 124.3 (75.6) | 155.8 (95.6) |

*CTAS, Canadian Triage and Acuity Scale; SD, standard deviation*

**This work is relevant, and it is essential to adhere to the journal's presentation format. The article can be published by organizing syntactical aspects and clarifying the presentation of the results. The statistical model appears appropriate, and publication is feasible with the suggested revisions.**
